# Cultured meat in the European Union: Legislative context and food safety issues

**DOI:** 10.1016/j.crfs.2024.100722

**Published:** 2024-03-16

**Authors:** D. Lanzoni, R. Rebucci, G. Formici, F. Cheli, G. Ragone, A. Baldi, L. Violini, T.S. Sundaram, C. Giromini

**Affiliations:** a Department of Veterinary Medicine and Animal Sciences (DIVAS), Università degli Studi di Milano, Via dell’Università 6, 29600, Lodi, Italy; b Department of Law, Politics and International Studies, Department of Excellence 2023-2027, Financed Through Funds of the Italian Ministry of University and Research, University of Parma, Via Università 12, 43121, Parma, Italy; c Department of Italian and Supranational Public Law, University of Milan, Via Festa del Perdono 7, 20122, Milan, Italy; d CRC, Innovation for Well-Being and Environment, Università degli Studi di Milano, 20122, Milano, Italy

**Keywords:** Novel food, EU regulation, Food market, Food safety, cellular food

## Abstract

The current food system, which is responsible for about one third of all global gas emissions, is considered one of the main causes of resource depletion. For this reason, scientific research is investigating new alternatives capable of feeding an ever-growing population that is set to reach 9–11 billion by 2050. Among these, cell-based meat, also called cultured meat, is one possible solution. It is part of a larger branch of science called cellular agriculture, whose goal is to produce food from individual cells rather than whole organisms, tracing their molecular profile. To date, however, cultured meat aroused conflicting opinions. For this reason, the aim of this review was to take an in-depth look at the current European legislative framework, which reflects a ‘precautionary approach' based on the assumption that these innovative foods require careful risk assessment to safeguard consumer health. In this context, the assessment of possible risks made it possible not only to identify the main critical points during each stage of the production chain (proliferation, differentiation, scaffolding, maturation and marketing), but also to identify solutions in accordance with the recommendations of the European Food Safety Authority (EFSA). Further, the main challenges related to organoleptic and nutritional properties have been reviewed.. Finally, possible future markets were studied, which would complement that of traditional meat, implementing the offer for the consumer, who is still sceptical about the acceptance of this new product. Although further investigation is needed, the growing demand for market diversification and the food security opportunities associated with food shortages, as well as justifying the commercialisation of cultured meat, would present an opportunity to position cultured meat as beneficial.

## Highlights

• Scientific research is investigating new alternatives that can feed an ever-growing population.
• Cultured meat, whose goal is the production of food from individual cells, is a great alternative.
• The European legislative framework for cultured meat reflects a precautionary approach.
• The cultured meat production chain must be analysed to identify possible safety critical points.
• Cultured meat could open new markets not replacing traditional ones.

## Graphical abstract

**Figure:**
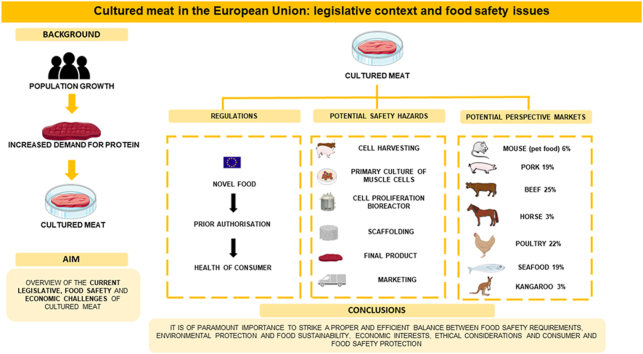

## 1 Implications

The current food system is characterised by a high environmental impact. For this reason, scientific research is investigating new alternatives capable of feeding a constantly and rapidly growing population. Among these, cultured meat could be a viable alternative. However, given the limited knowledge about this new technology and its recent introduction on the market outside europe, it is necessary to investigate not only the legislative aspects, but also the possible challenges in guaranteeing a safe product as traditional meat, investigating the possible future markets.

## 2 Introduction

The high impact of the food system on the environment is attracting increasing attention from the scientific community. The food system is a major cause of resource depletion and negative ecological footprint, being responsible not only for high land consumption, but also for the global eutrophication of oceans and fresh waters (Vermeulen et al., 2012; Lindgren et al., 2018). At the same time, as reported by Benton et al. (2021) and Dalin and Rodríguez-Iturbe (2016), over the decade (2006–2017), greenhouse gases (GHGs) production by the food system accounted for 28.9% (20.4–37.3%) of total global anthropogenic GHGs (52.0 ± 0.45%). More specifically, agriculture and land use were responsible for about 4.9 ± 2.5% of all GHGs, methane from ruminants and soil for about 4.0 ± 1.2%, fertilisers and manure for about 2.2 ± 0.7%, while transport, manufacturing and cooking were liable for 2.4 ± 4.8% (Benton et al., 2021). This scenario is expected to worsen, especially considering the steady growth of the world population, which is set to reach 9–11 billion people by 2050 (Röös et al., 2017). In parallel, there will be a dramatic growth in the demand for food, especially of animal origin, due to the fact that the Western diet, characterised by a high content of meat, fish and dairy products, has become a worldwide symbol of prosperity and economic growth, as well as an aspiration for newly urbanised countries (Bellet and Rushton, 2019). More precisely, as reported in the literature, food global demand will increase by 50% by 2030 and double by 2050, at which point it will be difficult to supply the demand without further worsening environmental health (Bellet and Rushton, 2019; Lanzoni et al., 2023). Therefore, considering the goal of feeding future generations, the promotion of socio-economic and environmental sustainability in the agri-food sector should be accompanied by the guarantee of a high level of food safety and consumer protection.

For this reason, traditional breeding is trying to move towards a more sustainable system, adopting strategies and technologies to achieve this goal. Among these, the use of feed matrices with a low environmental impact is a valid solution (Lanzoni et al., 2023a, 2024). In parallel, precision livestock farming is attracting great interest. It is, as reported by Tullo et al. (2019), defined as *‘the application of process engineering principles and techniques to livestock farming to automatically monitor, model and manage animal production*', the primary objective of which is to make livestock farming more economically, socially, and environmentally sustainable, through observation and, where possible, individual animal control. As demonstrated by Tullo et al. (2019), precision livestock farming has made it possible to reduce production risks and environmental side effects, such as the emission of pollutants into the air, soil and water, thus ensuring more sustainable livestock farming, safeguarding good health and high animal production.

Innovation and new technologies can therefore be considered valuable allies (Mouat and Prince, 2018; Siegrist and Hartmann, 2020; Martini et al., 2021; Treich, 2021). In this context, novel foods represent a great opportunity (Sforza, 2022).

Among the various alternatives, cultured meat is one possible solution. To date, it is known by several names, including cell based meat, in vitro, clean, synthetic, artificial meat, and lab- or factory-grown meat, although there is still no consensus on the correct terminology (Verbeke et al., 2015). Cultured meat, part of the broader branch of cellular agriculture science, represents the in vitro production of meat without the sacrifice of animals. More specifically cellular agriculture uses tissue engineering techniques, the aim of which is to produce food products (e.g. meat, fish, milk) tracing the molecular profile of traditional ones (Mouat and Prince, 2018; Eibl et al., 2021; Lanzoni et al., 2022).

Although cultured meat is of recent interest, the original idea has ancient roots. It first appeared in 1897 in a scientific novel entitled *Auf Zwei Planetem*, and then appeared in other accounts in the last century, as reported by Treich (2021). Later, in 1931, Winston Churchill criticised farming methods by introducing the subject of cultured meat with the following sentence: “*We shall escape the absurdity of growing a whole chicken in order to eat its breast or wing, by growing these parts separately in suitable soil. In the future, of course, synthetic food will also be used*” (Churchill, 1932; Ford, 2011). However, the development of cultured meat did not get much interest until the end of the 20th century. Starting in these years, before with the first patenting of the method of cultured meat production by Van Eelen et al. (1998), and then with the cultivation of goldfish meat by the National Aeronautics and Space Administration (NASA), cultured meat began to receive increasing interest (Benjaminson et al., 2002). The popularity of cultured meat, however, was only consolidated in 2013 with the presentation on live television of the first synthetic hamburger by Dutch researcher Mark Post (2014). From 2013 onwards, as reported by Chriki and Hocquette (2020), the number of scientific publications on cellular agriculture began to increase exponentially until the first marketing of the first cultured meat products in December 2020 in Singapore (Treich, 2021). To date, most of the research is conducted within startups located mostly in the USA and Europe, with a few others in Asia and Israel, financed by private investors (Treich, 2021; Cameron et al., 2019; Ye et al., 2022).

Given the rapid and growing interest, but above all, the possible future introduction of cultured meat in the European Union (EU) food market, it is necessary for scientific research to continue studying its possible critical points. While the production process has been described in the literature, it is incumbent upon us to investigate the critical points in the modulation of sensory and nutritional properties, deepening the issue of food safety along the entire production chain.

For this reason, the aim of the review is to provide an overview of the current legislative, food safety, technical, but also economic challenges of cultured meat. In particular, the paper intends to examine first of all the legislative regulations governing the marketing of this product, with a focus on the EU context. The pre-marketing authorisation procedure, established by the Novel Foods Regulation (European Commission, 1997), shows a close link between, on the one hand, legislative, political and ethical considerations and, on the other hand, scientific assessments. For this reason, this paper promotes an in-depth examination of food safety issues and the need to provide a comprehensive and careful analysis on this point. At the same time, the main critical points in the modulation of organoleptic and nutritional properties that can guarantee a product similar to the conventional one. Finally, the paper aims to illustrate possible future markets for cultured meat, with a focus on consumer acceptance.

## 3 How to regulate the marketing of cultured meat: the EU novel foods regulation between open challenges and political considerations

In recent years, innovation in the agri-food sector brought delicate and unprecedented challenges to food regulation (Ni and Lin, 2022). As highlighted in the recent European Commission Communication “Safeguarding food security and reinforcing the resilience of food systems” (March 23, 2022), new technologies - including New Genomic Techniques - are an indispensable tool for food security (European Commission, 2022). In this vast context, particular attention has been paid to the discipline of innovative foods, including both *per se* new foods, not existing before, and traditional food produced through innovative production procedures (Scaffardi and Formici, 2022). The entry into market of these so-called Novel Foods is usually subordinated to a prior authorisation based on a food safety risk assessment, delegated to scientific – generally independent – food authorities or agencies. This regulatory solution characterizes several countries, such as Canada, Australia, the EU, Israel, and the United Kingdom (FAO, 2022; Gross, 2021), where legislators have elaborated provisions specifically addressing the marketing of Novel Foods with the primary aim of ensuring a high standard of consumers’ health protection.

Due to its innovative (non-traditional) production process, cultured meat is mostly considered a Novel Food and should therefore follow the general rules established for these food products (Post et al., 2020). That's the case of Singapore, where the chicken nuggets and processed shredded poultry products containing cell-based chicken have obtained the world's first approval in 2020 (Singapore Food Agency, 2021). The authorisation has been granted by the Singapore Food Agency (SFA) following the procedure established by the regulatory framework for Novel Foods and Novel Food Ingredients, introduced in 2019 and disciplining the marketing of foods not having a history of safe use (Singapore Food Agency, 2019). According to this discipline, “substances with a history of safe use are those that have been consumed as an ongoing part of the diet by a significant human population (e.g. the population of a country), for a period of at least 20 years and without reported adverse human health effects” (SFA, 2023). Based on this definition, producers interested in placing on the market Novel Foods are required to submit safety assessments to the SFA, who is responsible for reviewing the studies. Precise documents, submitted by applicants, are periodically updated by the SFA as well as by the newly appointed Novel Food Safety Expert Working Group (Singapore Food Agency, 2023). Interestingly, in 2021 the SFA also promoted the Novel Food Virtual Clinics, “where novel food companies are able to proactively engage SFA at early stages of their research. With a clearer understanding of SFA's requirements at an early stage, companies can prioritise resources towards productive research directions which will minimise compliance costs and time” (Singapore Food Agency, 2022). Clear requirements and information, together with a cooperation and dialogue between SFA and private companies in an early phase, seem to have facilitated the authorisation procedure of cultured meat in Singapore: after the chicken nuggets, the SFA subsequently approved new formats of cultured poultry in 2021 and, more recently, in 2023, the use of serum-free media for the production of cultured meat, which represent a key advancement towards a completely slaughtering-free production (Good Food Institute, 2022). As affirmed by the SFA in several documents, with specific reference to cultured meat, the safety of the product is reviewed at three different levels, focusing on the I) production process (cell lines, culture media, reagents, toxicology etc.), the II) process and controls ensured (e.g. contaminants, adherence to good safety and hygiene practices) and, finally, on the III) final product which must meet the standards established by the national food regulation (e.g. presence of allergenic proteins) (Singapore Food Agency, 2022).

Similarly to Singapore, in the EU cultured meat falls under the Novel Foods Regulation (Reg. EU, 2015/2283). Although, at the time of writing, no authorisation procedures concerning cultured meat have been submitted to the EU Commission, the latter could undoubtedly be considered as a food “which has not been used for human consumption to a significant degree within the Union before May 15, 1997 (when the first EU novel food legislation entered into force), regardless of the dates of Member States' accession to the Union" (Art. 3, EU Reg. 2015/2283). The legislation also requires the new food to fit at least one of the ten categories listed in Art. 3, paragraph 2, letter a). The category n. VI, which refers to “food consisting of, isolated from or produced from cell culture or tissue culture derived from animals, plants, micro-organisms, fungi or algae”, clearly includes cultured meat, that therefore necessitates to obtain a pre-market approval in order to be marketed in the EU territory.

The current Reg. EU 2015/2283, entered into force in 2018, vastly reformed the previous authorisation procedure established by the outdated Reg. EC 258/97 (Pisanello and Caruso, 2018): now the procedure is entirely centralized both in the risk assessment and in the risk management phases (Volpato, 2022). The applicant is asked to submit a scientific dossier – including food safety studies – to the EU Commission that should provide a first formal check before appointing the European Food Safety Authority (EFSA) for the centralized and unique scientific risk assessment phase (maximum time length: 9 months, extensible for specific reasons) (Dall’Asta, 2022). On the basis of the EFSA opinion – which, by the way, is not binding – on the food safety of the product, the Commission is then in charge of the risk management phase, by preparing a draft implementing decision establishing the acceptance or the denial of the authorisation request and determining the possible marketing conditions – for example those concerning the labelling –. This draft decision needs the final approval of the Standing Committee on Plants, Animals, Food and Feed, where Member States’ representatives are seated (European Commission, 2023). Even if until now the decisions of the Commission have usually followed the assessment provided by EFSA, this last phase could be influenced by political and ethical considerations, differing from scientific evaluations focusing on food safety.

If the product obtains the authorisation, it is included in the so-called Novel Foods Union List (European Commission, 2023a) with a generic effect, meaning that all food business operators other than the applicant, interested in marketing the approved Novel Food, could place it on the market without submitting another application, unless a specific data protection and “secrecy” is granted (according to Article 26 of the Reg. EU, 2015/2283) (La Porta, 2021).

As clearly appears, the current legislative framework reflects a “precautionary approach” (Scaffardi, 2020) based on the assumption that innovative foods need a prior careful risk assessment in order to safeguard the highest standard of consumers’ health protection. EFSA consequently plays a significant and key role in the food safety procedure (Martini et al., 2020); for this reason, it comes with no surprise that this Authority is currently preparing to face possible authorisation requests concerning cultured meat: what emerges from initiatives such as the 2023 EFSA's Scientific Colloquium on “Cell culture-derived foods and food ingredients” is that guaranteeing a clear and fruitful communication with interested food business operators, institutions as well as consumers reveals extremely important when innovative foods are concerned (EFSA, 2023).

Cultured meat, in particular, seems to be a highly debated Novel Food in the EU territory, not only by the scientific community but also the civil society and, interestingly, by national policy makers and legislators. The case of Italy seems to be paradigmatic of the complex issues regulating Novel Foods entails: facing fears about the safety of cultured meat and its potential disruptive effect on traditional meat production systems (and cultural heritage). The Italian government has decided to propose, in March 2023, the adoption of a specific law banning food and feed made, isolated or produced from cell cultures or tissue cultures derived from animals, which clearly includes cultured meat (https://www.senato.it/leg/19/BGT/Schede/Ddliter/dossier/56933_dossier.htm). The legislative text, approved by the Senate (A.S. no. 651-A), was subsequently debated and approved by the Chamber of Deputies of the Italian Parliament on December 1, 2023 with law no. 172 (Official Gazette of Italian Republic, 2023). In the currently approved version, the production, use, sale, import, distribution and promotion of cultured meat (defined by the Government as ‘synthetic meat') will be banned. Recalling the precautionary principle recognised by Article 7 of EC Reg. 178/2002 and the possible risks not only for the health of consumers but also for the livelihood of the Italian agricultural sector, the Government's decision has opened up a lively political and academic debate (Formici, 2023) that also includes the possible future relationship between this national legislation and the aforementioned EU Novel Foods Regulation. As previously pointed out, the European regulatory framework is directly enforced in each Member State, so any future authorisation regarding cultured meat obtained at EU level would also have a binding effect in Italy, and the generic reference to the precautionary principle (already much debated in GMO cases) (Ragone, 2019) could prove insufficient to maintain the legitimacy of a national ban. Apart from the questions regarding the multi-level regulatory dimension, the Italian example shows how new foods – and in particular highly innovative products such as cultured meat – pose delicate legislative issues and prompt a regulatory discussion that is not entirely based on food safety considerations but reveals to be strictly interrelated with ethical, political and economic evaluations. Moreover, the Italian legislative proposal “comes as other governments are acknowledging the strategic importance of cultured meat towards both food security and global sustainability” (Bertero et al., 2023), thus underlining different regulatory and policy approaches to innovation in the agri-food sector. This situation, which could potentially lead to diverse political positions expressed by Member States within the EU Institutions, should prompt a renewed and careful debate, also concerning other aspects related to the marketing of innovative foods such as the information provided to the consumers and, therefore, the labelling of cultured meat: should this product be called “meat” and which information should be provided to consumers about its origin? These questions, which have already been at the center of a complex political discussion with regards to vegetal or vegan products such as burger or milk, need to be thoroughly explored (Sirsi, 2020).

The need to boost this regulatory analysis and debate seems to be extremely urgent, also considering recent relevant advancements concerning cultured meat. In the United States of America in June 2023 the Good Meat's cultured chicken obtained approval from the US Department of Agriculture (USDA), after having received in March of the same year a “no questions” letter from the US Food and Drug Administration (FDA) (Congressional Research Service, 2023). This landmark decision represents the signal of an evolving scenario and shows, at the same time, a different regulatory solution: while in the USA there is not a specific legislation dedicated to the entry into market of Novel Foods, in 2019 – with an anticipatory move – the two most relevant federal Authorities in the agri-food sector, the USDA and the FDA, outlined the marketing path of cultured meat through a specific inter-agencies agreement (FDA, 2019; Grossman, 2019; Sollee, 2022). Under this document, the FDA is in charge of the controls and assessment of the initial stages of production while the USDA is responsible for the oversight of the processing, labelling and packaging. The interested food business operators should promote a pre-market consultation firstly with the FDA, who evaluates the food safety information the company submitted and poses possible questions if doubts arise during the reviewing process. Moreover, the pre-market consultation process “allows developers to work with the FDA on a product-by-product basis and informs them of issues they must consider to produce safe food that does not violate the Federal Food, Drug and Cosmetic Act's requirements” (FDA, 2023). Notwithstanding the absence of a comprehensive legislation, the federal agencies’ agreement covered the procedural issues by providing indications on the consultation phase, on the information required as well as on the role attributed to the two interested federal authorities, in order to prompt a coherent and clear cooperation. Once again, particular attention has been paid to the cooperation between private actors and public agencies since the early development and research stage. The USA case, in which the marketing of cultured meat seems to be in an already well advanced phase, demonstrates the importance to provide regulatory answers and *ad hoc* procedural solutions to specific innovative foods, through the prior determination of rules and agreements. A similar approach could be identified also in Japan, for example, where the Association for Cellular Agriculture, a group of different stakeholders and institutions, guided by the Center for Rule-Making Strategies, has been founded with the final aim of “creating an industry guideline and a recommendation for new law to be implemented” (Miyake et al., 2023).

The different regulatory solutions promoted in several Countries as well as the political debate and the diverse approaches promoted in recent years (interesting are the cases of Israel and Chine, that boosted, also through public investments the research in alternative protein sources, FAO, 2022) demonstrate the importance not only of a comprehensive and accurate food safety assessment but also of an in-depth legislative debate on all the regulatory issues that innovative foods pose. In fact, we should consider that “the manner in which cellular meat is regulated will be a determining factor in the success of the product” (Sollee, 2022). The final aim of such debate is of fundamental importance: finding a correct and efficient balance between food security needs, environmental protection and food sustainability, economic interests, ethical considerations as well as consumers protection and food safety safeguard.

## 4 Cultured meat production: potential safety hazards

To date, cultured meat is one of the most hotly debated topics in science. It could be considered a more sustainable and safer product than traditional meat. However, as reported by Chriki and Hocquette (2020), this type of comparison is incomplete and sometimes biased, because nowadays there are no certain data, but only projections over the long term, which are difficult to compare with the data for traditional meat.

**Fig. 1:**
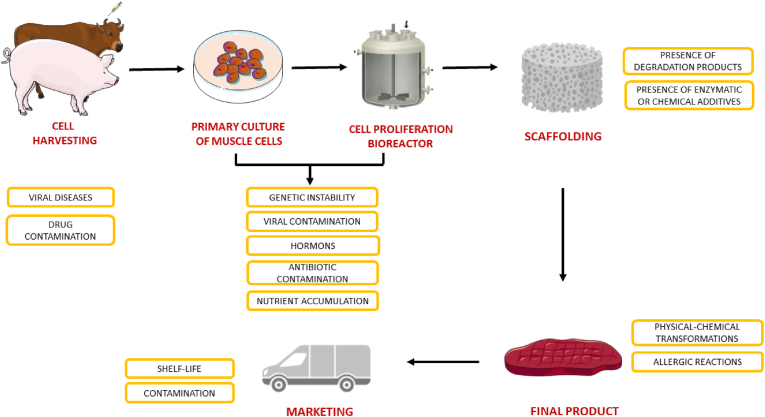
(Colour Figure) Description of all critical point on the production chain of cultured meat. The Figure was partly generated using Servier Medical Art, provided by Servier, licensed under a Creative Commons Attribution 3.0 unported license.

For sure, from an environmental point of view, the production of cultured meat will require less land and water use. More precisely, as reported by Haraguchi et al. (2022),1 kg of cultured meat (approximately 5 × 10^10^ cells), will require 50 L of water (used almost exclusively for the production of the culture medium), which is significantly less than the 550–700 L of traditional meat (Chriki and Hocquette, 2020; Santinello et al., 2023). Although this is well-established in the literature, it is also necessary to assess the quality of the water resulting from processing, the main waste product, the volumes of which are as yet unquantifiable. Indeed, as argued by Chriki and Hocquette (2020), waste media, containing growth factors, hormones and other chemicals, would represent a critical issue for environmental sustainability. However, scientific research is already investigating a green utilisation of such waste, promoting its use for the growth of microbial proteins for animal/human nutrition, as demonstrated by Haraguchi et al. (2022). In comparison with conventional livestock farming, as reported by Lynch and Pierrehumbert (2019), it will also be important to consider the impact of CO_2_, the main GHG produced in cultured meat production, which has a longer bioaccumulation period in the atmosphere than CH_4_, although it will need to be monitored over the long term for accurate analysis. Although based on long-term projections, environmental sustainability has been widely described in the literature. At the same time, as reported by Chriki and Hocquette (2020), the issue of safety is still a topic that need to be investigated. Proponents of cultured meat consider it a safer product than traditional meat, as it is produced in a closed and controlled environment (Chriki and Hocquette, 2020). However, it must be emphasised, that on a large scale the product will not be produced in the laboratory but on an industrial level, where it is impossible to completely eliminate possible risks, especially those due to human error. This is a common problem with plant-based protein products. Indeed, as reported by Banach et al. (2023), processing can introduce microbiological hazards such as *Staphylococcus aureus*, mainly through food handling (skin contact), or *Listeria monocytogenes* during processing, as it can be found in the processing environment. As reported by Jahir et al. (2023) and Stephens et al. (2018), to prevent this possibility, new research courses and skills will be required that can provide high levels of knowledge beyond the more traditional roles, including chemists, cell biologists, materials scientists, chemical engineers, skeletal muscle scientists, technicians, and food technologists. However, before showing the possible critical points in the production chain of cultured meat (Fig. 1), it is necessary to consider that traditional meat production is characterised by a high control system that must also be integrated for the cultured meat.

Cell Harvesting: It represents the first step in the production of cultured meat. It consists of a cell or tissue biopsy from a live animal or in the post-mortem period (Lanzoni et al., 2022). This step has been extensively studied in the literature with the aim of obtaining the greatest number of stem cells (satellite muscle cells) from a single animal (Post, 2012; Zhu et al., 2022; Guan et al., 2022). More precisely, the choice of cell sampling must not be random, but must take into account multiple variables, including age, sex and breeding conditions, in addition to genetic ones (Lanzoni et al., 2022). Indeed, during the animal's ageing, in addition to the decrease in the concentration of satellite cells in the muscle, the latter undergo a high number of mitotic divisions, thus maintaining their differentiation capacity for a much shorter period, compared to cells taken from young animals (Melzener et al., 2021). At the same time, as reported by Choi et al. (2021), male animals show a higher concentration of stem cells than females, due to the positive action of testosterone. Similar results are obtained with extensive compared to intensive housing, most probably due to the different type of diet (Lanzoni et al., 2022). In parallel, it is also essential to preserve animal welfare. For this reason, as argued by Melzener et al. (2021), cell harvesting should be done by needle biopsy, a less invasive technique than tissue harvesting. Furthermore, in order to reduce the number of donors, it is desirable to maximise the number of biopsies (maximum four in one session) from the same animal every three months, thus ensuring adequate recovery times for animal welfare (Melzener et al., 2021).

However, to date, the relationship between the health status of the animal on which the harvesting is performed and the potential introduction of biohazards into the cultured product has not yet been studied. As reported by Ong et al. (2021), the main food safety issues almost exclusively include the transmission and spread of infectious viral diseases. The latter can be transmitted in the following ways: I) In the form of free viral particles via faecal contamination of foodstuffs; obviously, this is not the case of cultured meat production; II) By transmission of infected cells to other hosts, e.g. hepatitis A, hepatitis E, bovine leukaemia virus (Ong et al., 2021). The latter mode of transmission represents a very delicate point, both because it is still unclear whether the cells of an infected animal undergoing biopsy are able to persist in culture, and also because of the risk of transmission of zoonotic diseases (e.g. bovine leukaemia virus) (Juliarena et al., 2017; Ong et al., 2021). Nevertheless, this risk can easily be circumscribed by a strict inspection of the source animals and biopsied cells/tissues for signs of infection.

Another possible risk at this stage concerns contamination by veterinary drugs, including antibiotics. They may be present as contaminants in the tissue used for cell harvesting and potentially present in the final product, causing adverse effects on human health (FAO & WHO, 2023). However, for this to occur, the drug must first be present in the sampled tissue and then in the cell culture throughout the production cycle, thus reaching the final product at a concentration that exceeds the maximum safe level. Nevertheless, this risk can easily be monitored either by using tests for the quantification of veterinary drugs on both the cell line and the final product, but especially by consulting the health data of the source animals at the time of tissue biopsy (FAO & WHO, 2023).

At this stage, it is also crucial to further investigate the controls for chemical contaminants, e.g. cryoprotectants used to store cellular models. Indeed, as reported by Ong et al. (2021), some cryoprotectants could be toxic if present in the final product. However, as pointed out by Savini et al. (2010) and Ong et al. (2021), cryoprotectants such as inulin, sorbitol, and dimethyl sulfoxide are already used in food processing to date and have proven to be safe. Despite this, to ensure total safety of cultured meat, it is expected that cryoprotectants will either be eliminated or diluted to very low concentrations and combined with washing of the final product (Ong et al., 2021).

Proliferation and Differentiation: The next step after cell harvesting is the isolation of satellite muscle cells and their culturing to first promote proliferation and then differentiation within bioreactors. At this stage, there are several critical points relating to food safety. As reported by Rosser and Thomas (2018), the number of cells required to produce 1 kg of protein from muscle cells is in the range of 2.9 × 10^11^ to 8 × 10^12^. To achieve these high numbers, the cells need to have a high proliferative capacity. However, this could lead to the formation of cancerous cells within the culture due to genetic instability, without being clearly identified within the cell cultures. Although such cells are harmless, as they are dead on consumption of the meat and therefore not incorporated viable within the body, they present a great challenges of acceptance for the consumer. For this reason, this needs to be further investigated to ensure the total absence of risk (Hocquette, 2016).

As previously reported, cells proliferate and differentiate in bioreactors, closed and controlled systems capable of providing all the stimuli the cells need to ensure their viability. In particular, cells need a constant supply of nutrients (carbohydrates, lipids, vitamins, minerals and micronutrients) provided through culture media. To date, identifying all the critical points at this stage is very complex due to the many different source of nutrients needed for different species, cell types and production steps (Burton et al., 2000; Yao and Asayama, 2017). For this reason, it is necessary to make a general overview of the possible risks at this stage. Nutrients present in culture media are commonly found in conventional foods. However, in culture products, a potential food safety problem would occur if in the formulation of a specific culture medium, one or more of these substances were present in the final product at concentrations that would be hazardous to the consumer (FAO & WHO, 2023). This could occur if the nutrient is accumulated abnormally or through cellular internalisation, or aggregation on structural material. In both cases, cells are able to metabolise the substance completely (FAO & WHO, 2023). To prevent this possible risk, different controls exist such as: I) use of minimum levels of nutrients that still allow cell growth, II) constant monitoring of cellular parameters (e.g. viability and morphology) as indicators of cellular damage, III) chemical analysis of the final cellular product to identify the nutrients present, whereby the maximum safe levels related to intake are already known for traditional foods (FAO & WHO, 2023).

To proliferate, cells not only need nutrients, but also additional secondary components that are essentials to provide cells with signals for their viability, replication and differentiation. These include animal serum, proteins, peptides, nucleic acids (micro ribonucleic acid (RNA) or miRNA, messenger RNA or mRNA), growth factors and hormones (FAO & WHO, 2023; Ong et al., 2021). For sure, to date, the greatest challenge in cultured meat production is to find a substitute for animal serum, in particular foetal bovine serum (FBS), that can replicate its characteristics while guaranteeing ethicality. Foetal bovine serum is a complex mixture of fatty acids, lipids, vitamins, carbohydrates, inorganic salts, growth factors, proteins, and more than 400 metabolites, which are essential for cell adhesion, growth, and proliferation (Lanzoni et al., 2022). Despite these many positive aspects, the production of FBS clashes with the ethicality promoted by cellular agriculture. In fact, it is taken by cardiac puncture from foetuses up to three months old from cows sent to slaughter, causing suffering and pain (Brunner et al., 2010). The exact amount of FBS produced and sold worldwide is unknown. However, it is estimated that about 800.000 L of FBS are sold annually, which corresponds to about two million foetuses sacrificed (Subbiahanadar Chelladurai et al., 2021). These data explain why its use for the production of cultured meat, besides being unethical, would in any case be unsustainable in the long run. Furthermore, FBS being an animal by-product could contain endotoxins, haemoglobins and other factors adverse to cell growth, as well as being a potential source of microbial contaminants (fungi, bacteria, mycoplasmas, viruses and prions) posing a health problem for the consumer (Brunner et al., 2010). Although, as reported by Chriki and Hocquette (2020), companies have already found a viable substitute to FBS (patent-protected), scientific research is investigating multiple substitutes. These include products of plant peptones, hydrolysates (yeast, rice protein, wheat and sericin), dairy by-products (whey proteins) and the use of extracts from microalgae (*Chlorella vulgaris* and *Spirulina maxima*) (Ho et al., 2021; Lanzoni et al., 2022). While the FBS problem is widely described in literature, the other components deserve further investigation. Indeed, the addition of proteins, peptides but also growth factors of animal origin, although essential to support cell growth, can introduce viral or prion contamination, as claimed by Jayme and Smith (2000). However, the same authors suggest how this problem can easily be curbed by using animal-free culture media, thus limiting the introduction of pathogenic organisms (Jayme and Smith, 2000). Possible substitutes may be plant-based products, as suggested by Chriki and Hocquette (2020). To date, companies are working hard to achieve this goal. One example may be BioBetter, an Israeli company founded in 2015, which has started to produce and market growth factors produced from tobacco plants for use in the production of cultured meat.

Particular attention must be paid to the use of hormones. Their excessive consumption can lead to imbalances and adverse human health outcomes, including pro-carcinogenic effects and reproductive toxicity, as argued by Jeong et al. (2010). For this reason, as early as 1996 (Council Directive 96/22 EC of April 1996), the European Union regulated the use of hormones in the traditional food chain, banning the use of certain hormonal substances such as testosterone, progesterone, zeranol, trenbolone acetate, melengestrol acetate, and oestradiol 17β, as they can remain as residues in the meat of treated animals following their slaughter (European Commission, 1996; Ong et al., 2021). This ban plays a fundamental role in food safety, being implemented not only for Member States but also for imports from third countries (European Commission, 1996). Possible solutions, as suggested by FAO & WHO (2023), could be the use of these substances at minimum concentrations that still allow the desired effect to be achieved, the use of product washing steps, and finally the implementation of safety and quality control measures (FAO and WHO, 2023).

Another problem related to cell proliferation phase concerns the use of antibiotics in the culture medium to prevent any contamination. Although the laboratory is a controlled environment with careful monitoring, it is difficult to stop any signs of infection, which is why they are added to the culture medium. However, it must be emphasised that these within the cell cultures will be added (when necessary) at lower concentrations than those used in traditional breeding and used almost exclusively in the early stages of production, where the cells will then be rinsed and purified, reducing the possibility of these being found in the final product, without the possibility of causing allergic reactions (Ong et al., 2021). At the same time, another possible problem concerns the development of drug resistance in the cells used. To prevent this phenomenon, as reported by Ramani et al. (2021), a possible solution could be the substitution of antibiotics with natural or synthetic antimicrobial peptides, lysins, bacteriocins, hydrolysed peptides, and biological extracts, which do not constitute a stress factor or create drug resistance. However, it is still necessary to document and record the use of antibiotics or a substitute, the type and concentration, increasing controls for human health safety (FAO & WHO, 2023; Ong et al., 2021).

At this stage it is also crucial to pay attention to chemical contaminants that are used in the medium, including antifoaming, pH buffers, culture media stabilisers as well as the accidental introduction of microplastics from water and the external environment (FAO & WHO, 2023). In this case, as suggested by FAO & WHO (2023) to safeguard consumer health, it is necessary to quantify the levels of these chemical contaminants at every stage, until the final product. In fact, such contaminants can occur at any stage of the production process, from harvesting to market.

Scaffolding: Tissue maturation only takes place if cells are provided with an environment in which they can first adhere and proliferate and subsequently differentiate. To enable this, scaffolds are used in the production of cultured meat, i.e. three-dimensional structures characterised by correct architecture, porosity, mechanical and chemical properties (Lanzoni et al., 2022; O'Brien, 2011; Seah et al., 2022). Considering the purposes of food engineering, they must be either biodegradable or edible or both, their structure being involved in the organoleptic properties of the final product (Lanzoni et al., 2022). Depending on the nature of the scaffold, different safety issues may arise for the end consumer. If the scaffold is designed to degrade, it is necessary that the material used and the degradation products are safe for human consumption, requiring a safety assessment typical of any food additive or ingredient (Ong et al., 2021). Where, on the other hand, the scaffold used is not designed to degrade and it is necessary to act via chemical or enzymatic dissociation, a characterisation of the additives used is required, as reported by Stephens et al. (2018), it is possible for them to persist within the final product.

Final Product: As a result of cell proliferation and differentiation, cultured meat is subject to the phenomenon of maturation before reaching the final stage. Although Olenic and Thorrez (2023) reported that cell lines do not undergo a true maturation process, Ramani et al. (2021) emphasised that maturation is influenced by electrical, mechanical factors, co-cultivation with other cell types, and growth factors. Despite this, at this stage, it is essential to implement controls to ensure quality and food safety. An important aspect to be assessed concerns the physical-chemical transformations that can be triggered in the final product. These types of transformations occur when the components present in the products interact with other substances leading to changes in the structure and/or sequence of the compound with the undesired appearance of reactive species harmful to human health (FAO and WHO, 2023). They can be induced by food processing as heat/chemical treatment (pasteurization, extrusion, smoking, and freeze drying) or during sterilisation in production processes (irradiation). In the first case, it is important to emphasise that the high temperatures reached during the cooking of high-protein foods, including cultured meat, can lead to the production of harmful substances such as heterocyclic aromatic amines, polycyclic aromatic hydrocarbons and advanced glycation, end-products from the Maillard reaction (Zhang et al., 2023). However, although to date there is no confirmation that this can also occur in cultured meat, as reported by Zhang et al. (2023), scientific research has rarely reported the presence of chemically hazardous substances in meat analogues, the latter of which are structured to resemble the typical structure of conventional meat. In the second case, although food irradiation has been approved in more than 50 countries, including Australia, Belgium, Brazil, Canada, China, Russia, South Africa, Thailand, the USA and Vietnam, there is no universal list of irradiable products, but varies from country to country with its own national regulations for labelling irradiated products (Madureira et al., 2022). In this context, the EU would seem to be curbing such treatment, having allowed only dried aromatic herbs, spices and vegetable seasonings to be irradiated through Directive 1999/3/EC (European Union, 1999). For this reason, to ensure the total absence of risk, in addition to evaluating and testing the physico-chemical transformations of the ingredients included in the formation of the final product, as suggested by the Food and Agriculture Organisation of the United Nations (FAO), it is necessary to have universally applicable food processing regulations (FAO and WHO, 2023).

One of the most important aspects to take into account in the final product concerns possible allergic reactions. Allergy to conventional meat is rare in adults and in most cases it is the alpha-gal syndrome, i.e. the immune system's reaction to a sugar molecule that could enter the bloodstream through a tick bite (Bryant, 2020). However, cultured meat, being molecularly similar to conventional meat, could trigger the same allergic reaction (Bryant, 2020). This doesn't represent the only risk. Indeed, during the production process of cultured meat, ingredients such as structural materials, media nutrients and modulators of cell function, whose adverse reaction is not yet known, may be introduced. This is an aspect in common with plant-based proteins and meat analogues (fungi-based) (Banach et al., 2023; Zhang et al., 2023). In fact, as reported by Banach et al. (2023) and Zhang et al. (2023), the increased prevalence of food allergies can occur in multiple ways: I) when proteins are removed from their natural matrix and incorporated in higher amounts into other constructs; II) by introducing proteins that are not normally consumed and cause primary sensitisation or show cross-reactivity to immunoglobulins of existing allergens; III) Triggered sensitisation towards new proteins can lead to cross-reactivity events towards foods that are currently not or rarely considered allergenic. For this, as reported by FAO & WHO (2023), it is necessary to increase controls at this stage including the selection of substances from non-allergic sources, use of minimum levels of these substances, quantification of potential residues in the final product and assessment of potential consumer exposure (FAO and WHO, 2023). Finally, as reported by Bryant (2020), a key aspect concerns clear labelling of the final product.

Marketing: The last and next step concerns the marketing and preservation of the final product. While on traditional meat, scientific research has adequately investigated the best strategies to maximise shelf life, on cultured meat it is still in its early stages. However, as reported in the literature by Gasteratos (2019), cultured meat, being prepared in sterile conditions, could be characterised by a longer shelf life than traditional meat while simultaneously reducing the costs of transport, refrigeration, and waste products. These aspects could also be favored by the fact that the production sites could be located closer to the consumer, compared to the farms (Tuomisto and de Mattos, 2011). The marketing of the product must take into account multiple aspects such as taste, colour and texture of the meat for the structure of even the final packaging (Siddiqui et al., 2022). Indeed, as previously reported, although bacterial contamination is possible during the production stages of cultured meat, it is crucial to note that bacterial infection can occur predominantly during transport and distribution due to poor quality packaging materials (Siddiqui et al., 2022). In this regard, the quality of the packaging material plays a key role in prevention, safeguarding consumer health. For this reason, Siddiqui et al. (2022) made an overview of packaging that can extend the shelf life of cultured meat while safeguarding food safety. In particular, the following packaging methods are taken into consideration: I) Modified atmosphere packaging, II) Vacuum packaging, III) Active packaging; the characteristics of which are briefly listed below.

I)Modified atmosphere packaging: This type of packaging prevents the oxidation of eme-proteins such as myoglobin and thus colour changes during storage (Siddiqui et al., 2022). More precisely, modified atmosphere packaging allows the atmosphere within the packaging system to be modified by reducing and/or removing oxygen inside the package from the top of the pack by modifying the gaseous atmosphere with nitrogen and carbon dioxide (Esmer et al., 2011; Siddiqui et al., 2022). At the same time, as reported by Djordjević et al. (2018), such packaging is able to reduce microbial growth; however, oxygen concentrations must be kept under control, as an absence of oxygen can lead to the development of anaerobic bacteria (Siddiqui et al., 2022).

II)Vacuum packaging: These packaging systems have been found to have positive effects on the shelf life of traditional meat (Lorenzo and Gómez, 2012; Devatkal et al., 2014; Brenesselová et al., 2015), which is why it can be assumed that they can also be used for cultured meat. Such packaging systems are effective in preventing colour change and the oxidation process by removing oxygen. The plastic material used for packaging the final product must ensure impermeability to prevent the absorption of oxygen from outside/inside the packaging system (Siddiqui et al., 2022).

III)Active packaging: These packages are of recent introduction to the market. They are defined as such because they are characterised by the presence of an active agent capable of interacting with the food contained in the packs, allowing them to increase their shelf life. Today, there are several types: I) The product to be consumed is coated with an edible material in such a way that the consumer can easily consume it while simultaneously ensuring a longer shelf life (Umaraw et al., 2020), II) The packages may contain an antioxidant agent or an oxygen scavenger inside them (Gvozdenko et al., 2022; Siddiqui et al., 2022), III) Introduction of an antimicrobial agent into the packaging system (Yildirim et al., 2018). Obviously, no reference is made to antibiotics, but natural compounds such as natural seeds to be integrated into the polymer. In this way, the packaging absorbs moisture from the meat and supports the release of antimicrobial compounds (Bahmid et al., 2021). An alternative solution could be the encapsulation of gases such as carbon dioxide and the incorporation of volatiles such as ethanol or essential oils that can inhibit bacterial growth (Siddiqui et al., 2022).

In the light of the above, it is clear that in order to prevent any form of contamination and ensure the safety of the final product for the consumer, it is necessary to follow the rules of good cell culture practice (GCCP) and good manufacturing practice (GMP). GCCP's primary objective is to promote the maintenance of high standards in the application of procedures and products for cell and tissue culture of animal/human origin and in parallel to encourage greater international harmonisation and standardisation of laboratory practices, quality control systems, safety procedures, recording and reporting, and compliance with laws, regulations and ethical principles (Bal-Price and Coecke, 2011). As just reported, among the main recommendations in addition to keeping a detailed record of all procedures carried out to identify possible contaminants in the final product, the GCCP recommends working under aseptic conditions, avoiding the use of antibiotics and controlling the quality of culture media (Bal-Price and Coecke, 2011; Ong et al., 2021). GMP refers to all those practices aimed at preventing the occurrence of hazards. More precisely, it involves widely applied food production practices that describe the sanitary operations and maintenance and related production and process controls that enable safe food production (Ong et al., 2021; Blanchfield, 2005). In parallel, alongside GMP, it is necessary to ensure Good Hygienic Practices, which are essential in the supply of food, applicable to industrial food production. In parallel, the Food Safety Management System must be applied to the future market for cultured meat. This system is not only responsible for food production, but also aims to transparently demonstrate how food safety has been planned and implemented throughout the entire production chain (Kafetzopoulos et al., 2013). Within the Food safety management system, an important role is played by Hazard Analysis and Critical Control Points, which is the most widely used international system for ensuring product safety and identifying possible microbiological, chemical and physical hazards that may occur during the production and processing of cultured meat, including quality assurance monitoring at every stage (not only for the final product, but also for all starting materials, solutions/products used, contamination procedures applied, and waste disposal/recycling) (Kafetzopoulos et al., 2013; Bryant, 2020). In this context, as reported by Bryant (2020), alongside the European regulation for the approval of in vitro products, a system of inspections at national level will be applied to ensure the wholesomeness of the final product, all under the monitoring of EFSA.

## 5 Organoleptic properties and nutritional profile: major challenges for cultured meat

One of the main challenges of cultured meat is to replicate the organoleptic, techno-functional and nutritional properties of conventional meat. Although, in some cases (e.g. Israel), cultured products are currently available to be marketed by specific companies, scientific research has a duty to explain and investigate possible critical points. Organoleptic properties (texture, colour and taste) play a key role in consumer acceptance (Broucke et al., 2023).

Texture: The texture of conventional meat is guaranteed by the maturation process, namely the reaction triggered only after the death of the animal (Lanzoni et al., 2022). More precisely, the cessation of oxygen leads to the accumulation of lactic acid and a lowering of pH that can activate several families of enzymes, that are essential for the breakdown of proteins and the subsequent tenderization of meat (Hocquette, 2016; Balasubramanian et al., 2021). However, to date, it is difficult to confirm that the maturation process also occurs for cultured products, due to reduced information in this regard. Certainly, texture would not be a critical point in products such as hamburgers or sausages, where the use of thin sheets of cultured cells would be able to replicate this characteristic (Broucke et al., 2023). In contrast, the production of whole cuts, due to their thickness, absence of blood and limited diffusion of nutrients and oxygen would make it difficult to replicate conventional texture (Broucke et al., 2023). To achieve this, various solutions such as cell stimulation in culture and co-cultures of myoblasts-fibroblasts-adipocytes have been adopted (Fraeye et al., 2020). At the same time, as reported by Broucke et al. (2023), additives such as proline, hydroxyproline, ascorbic acid in the culture medium can also be considered to alter the mechanical properties of the tissue or through the use of scaffolds that are essential for creating connective tissue. As reported by Cheng and Sun (2008), the tenderness of traditional meat is also due to its important water-retaining property. This is influenced by the formation of the actin-myosin bond, which is only created after the death of the animal. Although, cultured muscle fibres are characterised by the presence of actin and myosin, they are embryonic or neonatal forms and therefore would not be able to guarantee this feature (Thorrez and Vandenburgh, 2019). For this reason, although further investigation is needed, inexpensive solutions such as cellulose scaffolds or the use of water-retaining ingredients such as powdered egg white, fibre or starch may be applied to replicate this techno-functional property (Broucke et al., 2023).

Colour: The colour of the conventional product depends mainly on two basic parameters: myoglobin and iron concentration (Post and Hocquette, 2017). Laboratory-grown muscle fibres tend to be yellow, both because of the lack of myoglobin as it is repressed by cultured cells in the presence of oxygen, and because the main culture media contain minimal iron concentrations (Post and Hocquette, 2017). To achieve the traditional meat colour, it is possible to stimulate myoglobin production by reducing oxygen levels, increasing the iron content in the culture medium, and adding natural dyes directly to the final product (Fraeye et al., 2020). Another possible solution, as reported by Zhang et al. (2020), could be to add haemoglobin directly into the culture. This solution, however, would involve extracting haemoglobin either from animal blood, plant tissue or produced by microbial cells, which are expensive, time-consuming and therefore not feasible on a large scale (Zhang et al., 2020).

Taste: As reported by Balasubramanian et al. (2021), most of the chemical metabolites present in conventional meat are not only derived from muscle, but are the result of the animal's food intake and biological metabolism. These, together with the interaction of proteins, lipids, carbohydrates, nerves and blood vessels are responsible for the unique taste of meat (Hocquette, 2016). At the same time, it is crucial to consider how flavour also depends strongly on alterations in sugars, organic acids, peptides, free amino acids and degradation products that occur exclusively post-mortem (Broucke et al., 2023). Considering cultured meat, it is difficult to understand how these changes could occur in the absence of the animal being slaughtered. Therefore, to replicate a sensory profile similar to the traditional one, it is necessary to intervene directly on the cultured cells, particularly the adipose cells. In fact, as reported by Khan et al. (2015), Fraeye et al. (2020) and Broucke et al. (2023), fat is crucial in the aroma, juiciness and tenderness of the final product. For this reason, it is possible to adopt solutions such as co-cultures of muscle cells and adipocytes, the use of pre-adipocytes to increase intramuscular fat (Fraeye et al., 2020; Kuppusamy et al., 2020), the addition of carotenoids that can prevent the oxidation of fatty acids by limiting their rancidity and preserving the final flavour (Stout et al., 2020; Broucke et al., 2023), and choosing a biomaterial that enables the differentiation of a particular cell type, such as adipocytes (Post et al., 2020). Finally, it is feasible to add aromas directly to the final product that take consumer preferences into account. As reported by Zhang et al. (2020), possible options such as hydrolysates of soy sauce, defatted soy or mushroom protein when heated produce flavour compounds similar to those in beef.

The aim of culturing meat is also to replicate and also improve the nutritional profile of traditional meat.

Micronutrients: Among the main micronutrients in traditional meat, minerals (iron, selenium, zinc) and vitamins (vitamin B12) play a key role in maintaining human health (Hocquette, 2016). However, cells in culture are not able to synthesise them independently. For this, it is necessary to add these nutrients directly into the medium associated with binding and transport proteins to facilitate uptake by the cells (Broucke et al., 2023). Although such a practice is feasible, as argued by Chriki and Hocquette (2020), it needs to be investigated whether even in cultured products, these micronutrients provide the same positive effects for human health.

Lipid content: As previously reported, co-cultures with fat cells would allow the lipid fraction in cultured products. Although, traditional meat is characterised by a high lipid content, approximately 37 g per 100 g of meat are saturated fatty acids (Calder, 2018). For this reason, to increase the functionality of these new products, the production of particular polyunsaturated fatty acids (PUFAs) could be added to the disadvantage of saturated fatty acids, creating a functional and beneficial product for the consumer (Broucke et al., 2023).

Protein content: To date, characterising the protein profile of cultured products is complicated due to limited information. The primary goal remains to simulate the protein content of traditional meat (20–24 g per 100 g) (Calder, 2018). To achieve this objective, several strategies can be adopted. I) Use of electrical stimulation to encourage sarcomeres synthesis. This method, although very efficient, is characterised by a high cost and for this reason not applicable on a large scale (Thorrez and Vandenburgh, 2019); II) Optimisation of the culture medium by providing a higher content of free amino acids and resulting in a higher protein content. However, as argued by Broucke et al. (2023), although this approach would be more cost-effective, there is a need to further investigate the uptake of nutrients by cells and what changes they undergo once internalised. III) Use of edible or biodegradable protein scaffolds. This alternative, besides being economical and applicable on a large scale, would make it possible to modulate the amino acid profile of cultured products. More precisely, matrices rich in essential amino acids could be chosen for the formulation of these structures, opting for derivatives of plant origin or exploiting genetic engineering to produce transgenic organisms capable of synthesising desired amino acids (Stein et al., 2009; Broucke et al., 2023).

## 6 Cultured meat: potential perspective markets

**Fig. 2:**
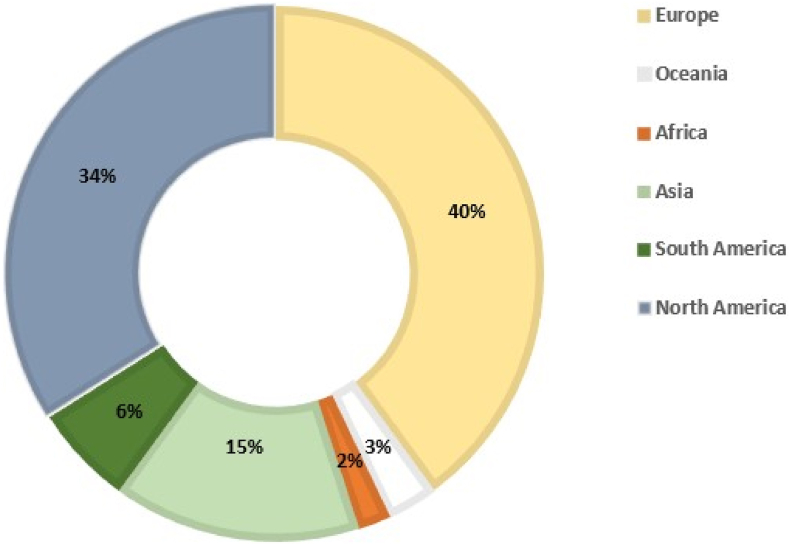
(Colour Figure) The global distribution of the cultured meat companies by 2021. Adapted by Ye et al. (2022).

**Fig. 3:**
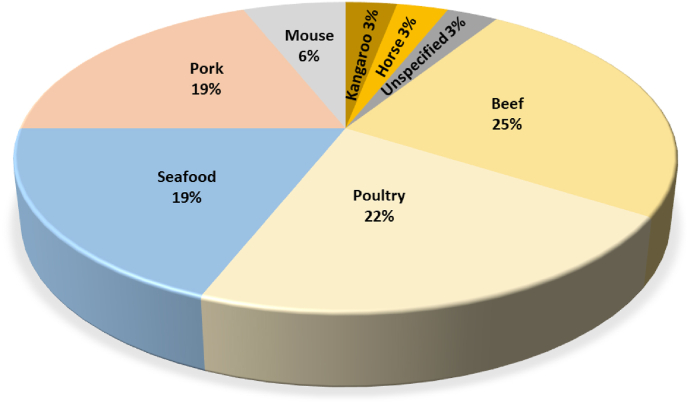
(Colour Figure) Primary meat focus for the cultured meat companies. Adapted from Choudhury et al. (2020).

The reasons that led to the discovery and development of the cultured meat sector are mainly related to sustainability and ethical reasons. In particular, as reported before, today's global population stands at 8 billion, a number that is set to grow dramatically by 2050, when the inhabitants on earth will reach 9–11 billion (Röös et al., 2017). At the same time, there will be an increase in demand for food, especially meat and dairy products. More precisely, in 2012, the FAO estimated that global demand for meat will reach 455 million tonnes by 2050, a 76% increase since 2005 (Bellet and Rushton, 2019; Lanzoni et al., 2022). All these reasons prompted the investigation of an as yet unknown market. As previously reported, enormous progress has been made in the production of cultured meat over the years. In 10 years alone, since the first cultured beef burger dated 2013, many start-ups (Table 1 and Fig. 2) with different production goals have emerged, as shown in Fig. 3.

**Table 1:** Consolidated companies operating in the field of cellular agriculture from 2015 to 2021.

Year	Company	State	Focus
2015	Integriculture	Japan	Cultured meat
	MosaMeat	Netherlands	Cultured meat
	SuperMeat	Israel	Chicken cultured meat
	Modern Meadow	New Jersey	Cultured meat
	Upside Foods	California	Beef, chicken, duck cultured meat
	BioBetter	Israel	Synthesis of growth factors for cultured meat
2016	Aleph Farms	Israel	Beef cultured meat
	Gelatex	Estonia	Scaffolding and microcarriers
	Because Animals	Canada	Cultured meat for petfood
2017	Nissin	Japan	Cultured meat
	Future Meat	Israel	Cultured meat
	BalleticFoods	California	Cultured meat
	Appleton Meats	Canada	Beef cultured meat
	Bio.Tech.Foods.	Spain	Cultured meat
	BlueNalu	Wales	Cultured sea-food
	Heuros	Australia	Cultured meat, synthesis of growth factors, media development, innovative packaging
2018	Fork&Good	New Jersey	Cultured meat
	denovoMATRIX	Germany	Production of microcarriers and scaffolds
	VitalMeat	France	Cultured meat
	Clear Meat	India	Cultured meat, FBS alternatives, synthesis of growth factors
	Meatable	Netherlands	Cultured meat
	New Age Meats	California	Pork cultured meat
	CubiQ food	Spain	Production of healthy fats
	Biftek.co	Turkey	Synthesis of growth factors
	Shiok Meats, Seafood, reinvented	Singapore	Cultured meat and sea-food
	Avant	Singapore	Cultured sea-food
	Innocent Meat	Germany	Synthesis of growth factors
	Higher Steaks	England	Cultured meat
	Cell Ag Tech	Canada	Development and production of sustainable cell-cultured sea-food
2019	Peace of Meat	Belgium	Chicken and duck cultured meat
	Orbillion	California	Beef Cultured meat
	Ivyfarm	England	Cultured meat
	LabFarm	Poland	Chicken cultured meat
	BioMilq	North Carolina	Cultured human milk
	MeaTech	Israel	Cultured meat
	Gaia Foods	Singapore	Cultured meat
	Brunocell	Italy	Cultured meat
	Artemys foods	California	Cultured meat
	TurtleTree Labs	Singapore	In vitro lactoferrin
	Vow	Australia	Cultured meat
	Mirai Foods	Switzerland	Cultured beef meat
	Matrix Meats	Ohio	Scaffolding
	OKPI	Russia	Cultured meat
	Joes Future Food	China	Pork cultured meat
	3DBT	England	Three-dimensional structures and serum-free medium
2020	Bluu Biosciences	Germany	Cultured seafood
	SiCell	China	Cultured meat
	BioMilk	Israel	Cultured milk
	Luyef	Chile	Cultured meat
	Novel Farms	California	Pork cultured meat
	Oxton Farms	England	Production of healthy fats
	Better Milk	Canada	Cultured milk
	Renaissance Farms	England	Cultured meat
	Umami Meats	Netherlands	Cultured meat
	MyoWorks	India	Scaffolding
	Mogale Meat CO	South-Africa	Cultured meat and Antelope cultured meat
	Meweri	Czech Republic	Pork cultured meat
	CellX	China	Cultured seafood, chicken and wagyu meat
2021	Another fish	Canada	Cultured seafood
	Meatafora	Israel	Cultured meat
	MicroMeat	Mexico	Cultured meat
	Bluefin Foods	California	Cultured bluefin tuna
	Quest Meat	England	FBS alternatives and primary cell lines
	Edge	USA	Synthesis of growth factors
	Anjy Meat	Croatia	Cultured meat
	JBS	Brazil	Cultured meat

More precisely, as Fig. 2 shows, the main companies are located for 40% in Europe (Croatia, Czech Republic, Estonia, France, Germany, Israel, Italy, Netherlands, Russia, Spain, Switzerland, Turkey, England), 34% in North America (America and Canada), 15% in Asia (China, India, Japan, Singapore, South Korea), 6% in South America (Argentina, Brazil, Chile, Mexico), 3% in Oceania (Australia) and 2% in Africa (South Africa) (Ye et al., 2022). Of these, as presented in Fig. 3, about 25% focus on beef, 22% on poultry such as chicken and duck, and 19% on pork and seafood such as fish and shrimp. In addition, two companies are investigating mouse meat as an alternative food for pets (Choudhury et al., 2020). Between 2015 and the beginning of 2020, the amount of capital invested in cultured meat companies (publicly disclosed), reached approximately $320 million. Approximately $243 million was allocated for the production of cultured meat from pork and beef, $50 million for seafood following the *business-to-consumer* business model, the main one in this sector. Alongside this, other business models have begun to emerge, such as *business-to-business*, the aim of which is the production of cell culture media, cell line generation, growth factors or, more generally, ingredients to be added to the culture medium (Choudhury et al., 2020).

However, to date it is difficult to go and study what the possible markets for cultured meat might be. There are no studies in the literature to refer to. In our opinion, cultured meat will not replace a market as complex as the traditional meat market, but will open up new markets to flank it, as reported below.

Over the years, intensive animal husbandry has undergone many changes that have resulted in a safe, nutritious and quality product for the consumer. As reported before, red meat is characterised by a high protein source. This value, combined with the lipid content, ensures a high energy intake (Lanzoni et al., 2022). In particular, meat is rich in saturated fatty acids, more specifically palmitic acid (C16:0) (about half), stearic acid (C18:0) (about one third) and lower concentrations of myristic acid (C14:0) and lauric acid (C12:0). Although stearic acid does not promote any effect on cholesterol, palmitic, myristic and lauric acid are responsible for raising blood cholesterol concentrations (Calder, 2018). At the same time, concentrations of PUFAs, recognised for their fundamental activity in maintaining human health, are low (Calder, 2018). In light of the above, a possible market could be the development of a “functional products” with a better nutritional and functional profile. Such an avenue would be pursued by adding cell-metabolisable nutrients to the culture medium, which would perform a positive function for the consumer. Although these products are not intended for vegans or vegetarians, as the origin is still animal (Mancini and Antonioli, 2020), they might be intended for a particular type of consumer.

Cultured meat could also find a place within certain religious communities: Jewish, Muslim, Hindu and Buddhist. The Jewish religion is characterised by Kashrut, i.e. a set of religious dietary rules, which prohibits the consumption of certain foods and requires others to be prepared in a specific way. To date, several issues concerning the Kashrut status of foodstuffs are still being examined with regard to cell-based products. Firstly, if products derived from animals prohibited by religious laws and considered Tareif, or forbidden for consumption by Jews, are themselves Tareif. Secondly, it must be determined whether these cell-based products, specifically those derived from mammals, are not considered meat products and should be handled as Parve (not classified as meat or a dairy product) as defined by Kashrut laws allowing them to be handled and consumed with dairy products. An example is the decision of the rabbinical organisation Thozar, which declared that meat products derived from embryonic stem cells taken from bovine blastocysts should be considered Parve, and as such eaten with dairy products. Such religious rulings play a crucial role in that they may substantially alter the diet of religious Jews (FAO and WHO, 2023).

For Muslims, the relevant question is if cultured meat is halal. As reported by Hamdan et al. (2018), based on Qur'anic scriptures, cultured meat can be considered halal if the cells used are derived from a halal slaughtered animal and if no blood or serum of animal origin is used in the production process. For this reason, it is very improbable that halal meat from pigs and other haram (not permitted) species will be approved. In fact, as reported by Bryant et al. (2019) out of 193 Muslims, 68% would eat cultured lamb or goat meat, 58% cultured beef, 49% cultured chicken, while only 28% cultured pork. In parallel, many Hindus interpret the principle of non-violence (ahimsā) as a requirement for vegetarianism (Bryant, 2020). This principle ensures that vegetarian Hindus consider cultured meat as a solution to avoid animal suffering. However, it is still unlikely that cultured beef will be accepted by Hindus, due to the sacred nature of this animal (Bryant, 2020). In fact, a study by Bryant et al. (2019), reported that out of 730 Hindus, 68% would eat cultured chicken, 65% goat, but only 19% beef. Finally, for Buddhist, 81% would eat cultured beef, 73% would eat cultured pork, 66% would eat cultured goat and 61% would eat cultured chicken (Bryant, 2020).

Another possible market is pet-food, an ever-expanding market worth around USD 100 billion a year. Trends in pet-food production towards so-called ‘human grade’ meat (meat perceived as of a quality suitable for human consumption), as well as potential changes in human dietary practices leading to fewer waste animal products, risk exacerbating the impact of pet-food, requiring a parallel increase in breeding and slaughtering mainly for the production of pet-food (Oven et al., 2022). All this has prompted pet owners to question what might be more sustainable alternatives, as reported by Oven et al. (2022). At the same time, pet feeding practices can raise ethical issues. Vegetarians and vegans face what has been termed the *vegetarian's dilemma* or the *animal lover's paradox* when deciding what to feed their pets (Oven et al., 2022). While they want products that meet the nutritional requirements of their animals, they also consider it a mistake to slaughter animals for food production. For this reason, the need and possibility of producing pet food using cellular agriculture technologies has arisen. To date, one of the main challenges concerns the final cost, given the fact that food intended for animals must be cheaper than food intended for human consumption. However, the possibility of using meat for which donor animals are not required due to the presence of immortal cell lines or the use of animals for biopsy whose breeding is less costly (e.g. mice, fish or invertebrates) may solve this critical point (Oven et al., 2022). In parallel, the application of cultured meat in the pet-food market would also require less regulatory burden, as it is generally less regulated than the human food chain (Oven et al., 2022). All these factors, coupled with the fact that pet food does not need to faithfully replicate existing products and thus less technologically demanding production, can be a springboard for the cultured meat market.

## 7 Cultured meat: consumer acceptance

Although scientific research is actively working to ensure the safety of cultured meat, consumer acceptance still remains a major challenge to overcome. The acceptance or rejection of this new product is generating conflicting opinions, also due to personal factors, also referred to as demographic predictors, such as age, gender, education, socio-economic status, and political orientation (Bryant and Barnett, 2020; Pakseresht et al., 2022). More precisely, as reported by Dupont and Fiebelkorn (2020), due to a lower level of food disgust, the younger part of the population would be more likely to consume cultured products. This difference is also visible between the male and female population. Although, Tobler et al. (2011) had reported that women were more willing to adopt ecological food diets, as shown by Slade (2018), Bryant and Barnett (2018, 2020), Mancini and Antonioli (2019) and Pakseresht et al. (2022), it would be men who showed a higher level of acceptance for cultured meat. As argued by Pakseresht et al. (2022), Grasso et al. (2019), education also plays a key role in acceptance, where more educated individuals are in favour of this new product. In fact, in support of this, as reported by Gómez-Luciano et al. (2019), in higher-income countries, cultured meat found greater favour than in lower-income countries, where people attribute status to greater meat consumption. Finally, political orientation also showed a division between supporters, the political left, versus opponents, the political right, distinguished by a more conservative feeling for cultural traditions (Bryant et al., 2019; Wilks et al., 2019).

Despite these predictive factors, as reported below, there are many different reasons for the rejection or acceptance of cultured meat.

### 7.1 Opinions against accepting cultured meat

Food*neophobia*and unnaturalness: Food *neophobia* has been identified as a major predictor of novel food rejection in Europe, Asia and America (Pilner and Hobden, 1992; Bryant and Barnett, 2020). This can be attributed to *food fussiness*, the strong preference for food prepared and served in a specific and familiar way, over a product that is often considered unnatural (Grasso et al., 2019). This is coupled with the unnaturalness of these new products leading to the rejection of cultured meat (Bryant and Barnett, 2020).

Disgust: Linked to unnaturalness and *food neophobia* is certainly the perception of disgust, a much stronger feeling in Western cultures, as reported by Siegrist et al. (2018). However, it is interesting to note that several researches have reported that cultured meats elicit less disgust than GMOs and insects, but more disgust than plant-based products (Dupont and Fiebelkorn, 2020). This difference is probably due to the familiarity of these products. At the same time, the disgust is not only related to the sensory profile, but should also be understood as a moral one. This distinction plays an important role as such objections may be surmountable in the long run, when cultured meat is likely to be a more well-known product (Bryant and Barnett, 2020).

Safety: As reported by Siegrist and Sütterlin (2017), it is also common for a proportion of consumers to have doubts about safety, in particular due to uncertainty about the long-term health effects of cultured meat. However, although this attitude seems to decrease in the presence of additional information about the entire production process (Bryant and Barnett, 2020), to date, as also reported by Chriki and Hocquette (2020), it is impossible to know what the harmful effects on human health might be, as cultured meat is a newly developed product.

Nutritional aspects: As initially reported by Laestadius and Caldwell (2015) and subsequently confirmed by Bryant and Barnett (2018) and Mancini and Antonioli (2019), sceptical consumers consider cultured meat an unhealthy and nutritionally inferior product compared to traditional meat. This aspect, which is also common for plant-based products, is most probably to be related to the artificial aspect and thus the non-naturalness of these new technologies (Bryant and Barnett, 2020).

### 7.2 Opinions in favour accepting cultured meat

Sustainability: Sustainability is considered to be the first advantage in the acceptance of cultured meat. As reported by Tuomisto (2019), consumers keen to support cultured meat promote its benefits on research use, such as reduced land use, less water wastage and reduced GHGs. This is reinforced with additional information demonstrating the low environmental impact compared to conventional meat (Mancini and Antonioli, 2020).

Ethics and morality: Cultured products are considered to be more ethical and moral as they would avoid suffering (confinement in confined spaces, probable bad breeding conditions) and the slaughter of animals, an advantage considered crucial for these new products (Van der Weele and Driessen, 2019). This aspect also plays a key role in the vegetarian's dilemma, using cultured meat for pets unable to follow a vegetarian diet, as previously reported (Oven et al., 2022).

Healthiness and safety: The potential benefits of consuming cultured meat could be both a healthier product, including a reduction in saturated and monounsaturated fatty acids in favour of PUFAs (Laestadius and Caldwell, 2015; Bryant and Barnett, 2018), as previously reported, and a safer product (Bryant and Barnett, 2018, 2020). However, as shown by Bryant and Barnett (2020), such benefits tend to be less commonly perceived than ethical and environmental issues, and are only identified after being solicited. It is important to note that safety has previously been identified as a parameter for the rejection of cultured meat. It is likely that safety, as a factor in support of this new product, is associated with those countries where conventional meat production has been regularly marked by deficiencies and diseases, as reported by Zhang et al. (2020a).

World Hunger: In parallel, as reported by Laestadius (2015), cultivated products are seen as an important means of feeding the world's population. In support of this, in the survey conducted by Mancini and Antonioli (2019), participants identified this benefit as one of the most common, only after sustainability and ethicality.

While scientific research has focused so much on consumer perception, it is also important to consider the opinions of stakeholders. As reported by Freeman (1994), stakeholders are groups or individuals that can influence or are influenced by the achievement of specific economic goals. These groups may include employees, suppliers, shareholders but also public groups such as governments and communities that provide infrastructure and indirectly regulate market activities (Clarkson, 1995). Among the main positive aspects called for by stakeholders, as reported by Amato et al. (2023), animal welfare and environmental protection are certainly the most important. However, these are associated with the belief that the technology industry will bring drastic changes to traditional agriculture, negatively impacting biodiversity and agricultural landscapes where animals are no longer needed (Amato et al., 2023). Another important category concerns the economic aspect, which involves conflicting opinions. While the positive aspects relate to better efficiency in manufacturing, the diversification of production, the establishment of new sectors and the creation of new job opportunities, one of the main negative aspects, expressed by stakeholders, concerns the possibility of monopolisation of new markets by large companies at the expense of smaller ones, especially in the early stages of market development, where large investments would be required (Newton and Blaustein-Rejto, 2021; Böhm et al., 2018; Amato et al., 2023). In parallel, stakeholders consider cultured meat to be a healthier and more nutritious alternative, with less hormones, antibiotics, animal-derived bacteria and easily modulated, which would allow the creation of specific functional products for certain consumer classes, as previously reported (Woll and Böhm, 2018). At the same time, however, the issue of safety is still unclear, with a split in stakeholder opinion, suggesting a more thorough investigation of this delicate topic (Amato et al., 2023).

Although the above aspects are crucial in the acceptance or rejection of cultured meat, the still uncertain price plays perhaps the most important role in determining the long-term success of this product. To date, there is much contradiction with respect to the economic issue. In fact, although Bryant and Barnett (2018), and Laestadius and Caldwell (2015) identified a probable high cost as a major barrier to purchasing cultured meat, greater even than *food neophobia*, in the study conducted by Mancini and Antonioli (2019), about 23.2% of the interviewees were willing to pay more for this new product, about 20.8% were ‘maybe' willing, while 26.7% were not willing to pay a premium (those who were not willing to try cultured meat). These percentages may increase if a sensory experience is associated, as reported by Rolland et al. (2020).

But what is the likely cost of cultured meat? According to the study reported by Garrison et al. (2022), cultured meat produced on a large scale could be produced at a cost of 63 $ for kg, where the major production costs are associated with the culture media (especially hormone production), bioreactors/equipment and labour, accounting for about 87% of the final cost (55 $ for kg). However, this cost estimate may never be reached as it will require huge technological advances to be realised. For this reason, possible solutions must be found to lower prices. First of all, low-cost culture mediums need to be investigated, which would lead to a substantial reduction in the price; secondly, used equipment from the medical and pharmaceutical industry could be used (Garrison et al., 2022). Although, great progresses have been made since 2013, where the cost of production was 2.3 million $ per kg (Post, 2014), it is unthinkable that cultured meat could be considered an affordable product for everyone, but it could be considered a niche product, especially in those economically developed countries such as Western Europe and the United States, confirming earlier reports on parallel markets for this new technology (Garrison et al., 2022).

In general, it is important to emphasise how different surveys lead to different results. For example, in the work reported by Wilks and Phillips (2017), the average acceptance rate for cultured meat was 63.5%, while the same parameter, identified by Hocquette et al. (2015), varied between 5 and 11%. These results, as pointed out in our previous review (Lanzoni et al., 2022) are discordant due to the population and sample considered, as well as the structure of the questions. Most probably, as also suggested by Post (2014), the acceptance of this product by future consumers will remain speculative until the product will be on the market.

## 8 Cultured meat: future perspectives

The research of cultured meat is an ever-expanding field, the literature is growing rapidly and global escalation seems imminent, although there are still many doubts that need to be cleared in the future. In terms of environmental benefits, cultured meat will face the challenge of being the second most energy-intensive source of protein during its production; a challenge that can be overcome by scaling up its production, as reported by Deliza et al. (2023). Achieving this goal would allow this new product to be classified as environmentally friendly. As reported by FAO & WHO (2023), the issue of safety has already been extensively discussed, identifying all possible risks at every stage of production, up to the final product. This approach will have to be kept alongside the control systems typical of the traditional supply chain in order to guarantee total safety. Nevertheless, before cultured meat reaches consumers' tables, large-scale follow-up studies will be needed, identifying new possible critical points and solutions, which in a narrow market would not be identifiable (Zhang et al., 2023). This step will have to be implemented especially in those countries where cultured meat struggles to find favour with food safety authorities and policy leaders, taking Israel, the first country to regulate the human consumption of cultured meat, as a model. Clear regulation would certainly meet with a greater consensus of public opinion, some of which is currently unfavourable. For this reason, as reported by Berry et al. (2017), it is necessary to implement a multidisciplinary approach involving more diverse fields (scientists, designers, marketing experts, psychologists, sociologists) in order to better understand consumer concerns and significantly increase acceptance, while optimising the design of new products.

As previously reported, cultured meat will not replace a market as complex as the traditional meat one, but will open new commercial windows alongside it. However, the commercial starting point should replicate those of existing meats both for acculturation purposes and for initial market penetration, and then facilitate market segmentation at a later stage (Deliza et al., 2023). In parallel, the high and flexible technological nature of cultured meat would also allow for a greater focus on customer needs during product and packaging development, further customising flavour, nutritional and sensory properties.

Ultimately, the growing demand for market diversification and the food security opportunities associated with food scarcity, as well as justifying the marketing of cultured meat, would present an opportunity to position cultured meat as beneficial.

## 9 Conclusion

In conclusion, cultured meat could represent a viable alternative to proteins of animal origin, whose future introduction into the market needs clarity, especially from a regulatory perspective. The current European legislative framework for cultured meat reflects a precautionary approach based on the assumption that such innovative foods require thorough prior risk assessment in order to safeguard consumer health. This assessment must be carried out at every stage of the production chain, more precisely from cell harvesting and related proliferation and differentiation, to the marketing of the final product, identifying possible solutions in accordance with EFSA warnings. A clear regulation, coupled with a safe and transparent production process, would allow both to increase the consensus of public opinion, still today divided on the positive and negative aspects, and the development of future markets, which will most likely parallel that of cultured meat. Although these aspects must continue to be investigated in order to ensure a safe product for the consumer, the growing demand for market diversification and the food security opportunities associated with food scarcity, in addition to justifying the commercialisation of cultured meat, would present an opportunity to position cultured meat as future food.

## Ethics approval

Not applicable.

## Financial support statement

•PRIN: Progetti di Ricerca di Rilevante Interesse Nazionale – Bando 2022. Prot. 20229S4T77, Project “Cellular agriculture for sustainable and innovative food production - “CELLtoFOOD”.
•PRIN: Progetti di Ricerca di Rilevante Interesse Nazionale - Banach et al., 2023. Prot. 2022EPRMH9, Project “The Future of Food, the Food of the Future”.
• PRIN: Progetti di Ricerca di Rilevante Interesse Nazionale - Banach et al., 2023, Prot. P2022B9P5H, Project “Coping with Climate Change. Method, Reasons and Procedure for Science Based Policy Making”.
• Project funded by the EU – NextGenerationEU – Piano nazionale di Ripresa e Resilienza (PnRR) – Missione 4, Componente 2, Investimento 1.3. – Avviso n. 341 del 15 marzo 2022 del Ministero dell’Università e della Ricerca. Numero identificativo: PE00000003, Decreto Direttoriale MUR n. 1550 dell’11 ottobre 2022 di concessione del finanziamento, CUP D93C22000890001, “On Foods – Research and innovation network on food and nutrition sustainability, safety and security – Working On Foods”.
• Jean Monnet Module (JERASMUS-JMO-2023-Module), Prot. FJ_MONNET23VIOL_01. Project “Feeding Future Generations Sustainably: Legal Challenges and Scientific Innovation” (FeedInn).
• Project funded under the National Recovery and Resilience Plan (NRRP), Mission 4 Component 2 Investment 1.3 - Call for proposals No. 341 of March 15, 2022 of Italian Ministry of University and Research funded by the European Union – NextGenerationEU; Project code PE00000003, Concession Decree No. 1550 of October 11, 2022 adopted by the Italian Ministry of University and Research, CUP D93C22000890001, Project title “ON Foods - Research and innovation network on food and nutrition Sustainability, Safety and Security – Working ON Foods”.

## CRediT authorship contribution statement

**D. Lanzoni:** Conceptualization, Writing – original draft, Writing – review & editing. **R. Rebucci:** Writing – review & editing, Writing-review. **G. Formici:** Conceptualization, Writing – original draft, specifically Paragraph 1, Writing – review & editing. **F. Cheli:** Conceptualization, Writing – review & editing. **G. Ragone:** Writing – original draft, specifically Paragraph 1, Writing – review & editing. **A. Baldi:** Writing – review & editing, Writing-review. **L. Violini:** Writing – review & editing, Writing-review (specifically Paragraph 1). **T.S. Sundaram:** Writing – review & editing. **C. Giromini:** Conceptualization, Writing – review & editing, Supervision.

## Declaration of competing interest

The authors declare that they have no known competing financial interests or personal relationships that could have appeared to influence the work reported in this paper.

## Data Availability

No data was used for the research described in the article.
